# Emerging Roles of UDP-GalNAc Polypeptide N-Acetylgalactosaminyltransferases in Cardiovascular Disease

**DOI:** 10.14336/AD.2024.0308

**Published:** 2024-03-08

**Authors:** Liwei Guo, Lulu Zhou, Pengcheng Wei, Shijie Li, Shanqing He, Duan Li

**Affiliations:** ^1^School of Forensic Medicine, Xinxiang Medical University, Xinxiang, Henan, China.; ^2^School of Basic Medicine, Xinxiang Medical University, Xinxiang, Henan, China.; ^3^Xinxiang Key Laboratory of Metabolism and Integrative Physiology, Xinxiang, Henan, China.; ^4^Henan Key Biological of Biological Psychiatry, Xinxiang Medical University, Xinxiang, Henan, China.; ^5^Department of Cardiovascular Medicine, Beijing Shijitan Hospital, Capital Medical University, Beijing, China

**Keywords:** UDP-GalNAc polypeptide N-acetylgalactosaminyltransferases, Mucin-type O-glycosylation, O-linked N-acetylgalactosamine, Cardiovascular disease

## Abstract

UDP-GalNAc polypeptide N-acetylgalactosaminyltransferases (GalNAc-Ts) catalyze mucin-type O-glycosylation by transferring α-N-acetylgalactosamine (GalNAc) from UDP-GalNAc to Ser or Thr residues of target proteins. This post-translational modification is common in eukaryotes, yet its biological functions remain unclear. Recent studies have identified specific receptors in the heart and vascular wall cells that can be mucin-type O-glycosylated, and there is now substantial evidence confirming that patients with various cardiovascular diseases (CVDs), such as heart failure, coronary artery disease, myocardial hypertrophy, and vascular calcification, exhibit abnormal changes in GalNAc-Ts. This review aims to highlight recent advances in GalNAc-Ts and their roles in the cardiovascular system, intending to provide evidence for clinical treatment and prevention of CVDs.

## 1. Introduction

Protein glycosylation is the covalent attachment of sugars or glycans (which are multi-sugar polysaccharides or complex oligosaccharides) to select residues of target proteins [1]. As one of the most common post-translational modifications, glycosylation expands an organism’s proteome beyond what is encoded by the genome and affects protein function, subcellular localization, and stability [2]. Protein glycosylation can be broadly classified into N-linked and O-linked glycosylation, and O-linked glycosylation is subdivided into two main types: mucin and non-mucin types [3]. In the mucin type, the attached monosaccharide residue is N- acetylgalactosamine (GalNAc). While in the non-mucin type, the attached residue can be N-acetylglucosamine (GlcNAc), fucose, mannose, glucose, xylose, or galactose [4, 5].

This review mainly focuses on mucin-type O-glycosylation, which is initiated by adding GalNAc to Ser or Thr residues of proteins in the Golgi[6]. This process in mammals is initiated and regulated by a large family of 20 UDP-GalNAc: polypeptide N-acetylgalactosaminyl-transferases (GalNAc-Ts) (EC2.4.1.41) [7]. The cardiovascular system is an intricate and well-organized network in which glycosylation plays a crucial role. Growing evidence supports the different roles of GalNAc-Ts in modulating cell signaling pathways and targeting proteins that are critical for maintaining cardiovascular homeostasis, for example: 1) Mutations in the GalNAc-Ts are linked to modifications of plasma lipid concentrations, which in turn increases the susceptibility to cardiovascular diseases (CVDs) [8, 9]. 2) O-glycosylation of low-density lipoprotein receptor (LDLR)-related proteins substantially increase their lipid binding and uptake [10, 11]. 3) Changes in mucin-type O-glycosylation pattern of the cell surface expressed G protein-coupled receptors (GPCRs) can potentially serve as biomarkers for cardiac hypertrophy and heart failure(HF) [12, 13]. 4) GALNT1 affects the proteases a disintegrin and metalloprotease with thrombospondin type1 motifs (ADAMTS1) and ADAMTS5, cleaving the proteoglycan versican and other extracellular matrix proteins during valve development [14]. 5)A series of studies by our group revealed novel aspects of the function of GalNAc-T3 in endothelial damage and vascular calcification [15-18].

Recent emerging evidence highlights the importance of GalNAc-Ts in the cardiovascular system [17, 19, 20], however, their biological functions are not yet fully understood. This review focuses on the recent advances in GalNAc-Ts and their roles in the cardiovascular system, intending to provide evidence for clinical treatment and prevention of CVDs.

## 2. The biosynthesis of mucin-type O-glycosylation

Mucin-type O-glycosylation is an evolutionarily conserved protein modification characterized by the initial addition of a GalNAc monosaccharide to the hydroxyl group of Ser or Thr residues[21]. After the initial addition of GalNAc, the extension of the sugar chain occurs in a stepwise manner, yielding several higher-order glycan structures. It can be divided into four main processes: initiation, core extension, elongation, and capping[22]. This section will detail the biosynthesis process of mucin-type O-glycosylation.

**Figure 1.**
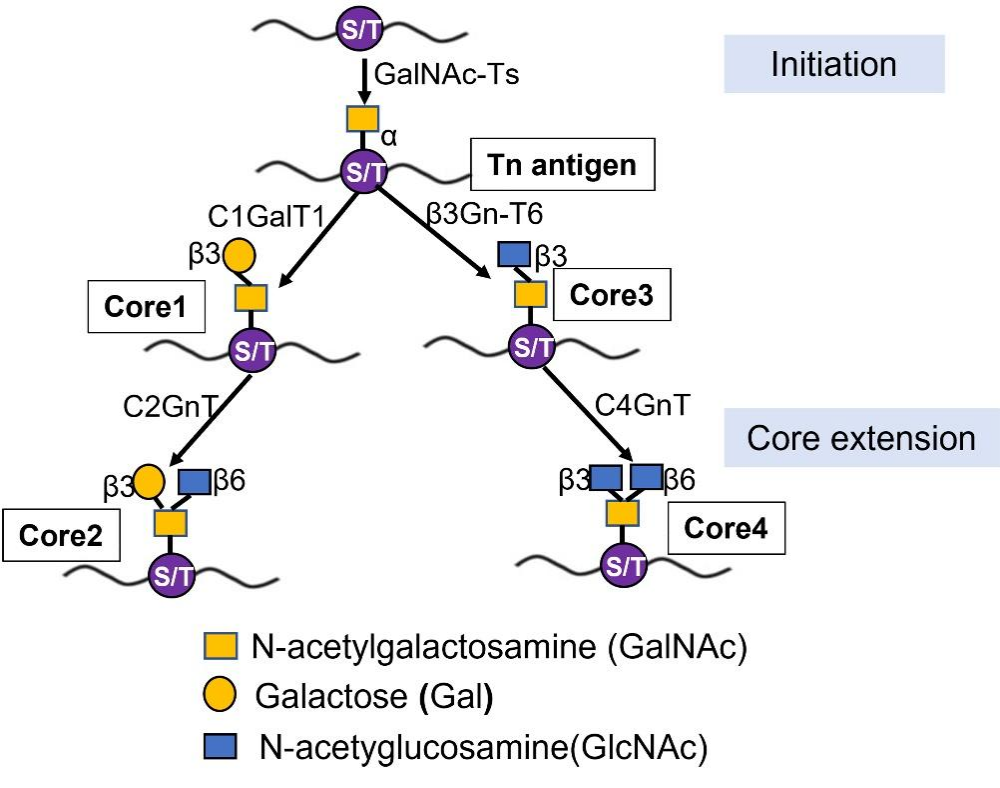
**Biosynthesis of core1 and core3 derived mucin-type glycan**. Mucin-type O-glycosylation is initiated by GalNAc-Ts forming the Tn antigen. The Tn antigen can be elongated by C1GalT1 which catalyzes the addition of a Gal residue, leading to the biosynthesis of the core 1 structure. Tn antigen forms core 3 structure catalyzed by β3Gn-T6. Cores 1 and 3 are transferred by C2/C4GnT to form core 2 and 4, respectively.

The first step in mucin-type O-glycosylation is the linking of UDP-GalNAc to Ser or Thr residues of target proteins, forming the Tn antigen [4]. This step is essential for the biosynthesis of O-GalNAc because it determines the amino acid position on the receptor protein. After synthesizing the Tn antigen by GalNAc-Ts, core extension takes place by different glycosyltransferases, of which core 1 to core 4 are the most common. Core 1 structure is formed when Galactose (Gal) is added to the Tn antigen by core 1 β1,3-galactosyltransferase (C1GalT1), resulting in the Galβ1-3GalNAc-α-O-Ser/Thr structure. This enzymatic activity is dependent on the action of the core 1 β3-Gal-T-specific molecular chaperone COSMC [23]. At the same time, the Tn antigen can also form a core 3 structure catalyzed by β1,3-N-acetylglucosaminyltransferase 6 (β3Gn-T6). Core 1 and 3 structures are branched by core 2/core 4 β-1,6-N-acetylaminotransferase (C2/C4GnT) to form core 2 and 4, respectively (as shown in Fig. 1).

The core 1-derived O-glycans are widely expressed in a variety of cells such as glomerular endothelial cells of the kidney[24], hematopoietic cells of the liver[25], and neural cells[26]. On the other hand, core 3-derived O-glycans are primarily restricted to the intestinal mucosa and salivary glands[27]. Whereas core 2 to 4 structures are exclusively present in the proteins and secretions of the mucosa[28].

## 3. UDP-GalNAc: polypeptide N-acetylgalacto-saminyltransferases

Mucin-type O-glycosylation is initiated by a family of glycosyltransferases known as the GalNAc-Ts [29]. Over 20 isoenzymes of GalNAc-Ts have been reported so far, with diverse biological roles[5, 30]. Understanding the structure of GalNAc-Ts and their preference for peptides is critical for elucidating their biological functions.

The members of the GalNAc-Ts family are type II transmembrane proteins that share common structural features: a short cytoplasmic N-terminal tail, followed by the type II transmembrane domain, a first catalytic domain (subdomain A) containing a substrate and a manganese binding site, a second catalytic domain (subdomain B) containing the Gal/GalNAc motif responsible for the binding of UDP-GalNAc, and a C-terminal lectin domain in Golgi apparatus that is connected by a flexible linker [31]. The distance and orientation between the catalytic and lectin domains determine the specificity and binding affinity and differ significantly across the isoforms [32].

It is now known that the GalNAc-Ts can be classified into three subfamilies based on their preference for peptides: 1) Glycopeptide/peptide-preferring isoforms (e.g. GalNAc-T1 and GalNAc-T2); 2) (Glyco) peptide-preferring isoenzymes (e.g. GalNAc-T4); and 3) Strict glycopeptide-preferring isoenzymes (e.g. GalNAcT7 and GalNAc-T10) [33]. Identifying glycosylated substrates is crucial in understanding the role of the GalNAc-Ts [34]. Most isoenzymes of GalNAc-Ts are capable of glycosylating common acceptor substrates, especially those that contain the (Thr/Ser)ProXPro motif, where ‘X’ usually stands for a small hydrophobic residue, suggests that they may serve redundant functions [35]. However, recent studies have demonstrated that several GalNAc-T isoenzymes are highly specific for particular protein substrates. For instance, GalNAc-T2 was found to specifically glycosylate ApoC-III[36], and GalNAc-T11 was reported to be specifically involved in glycosylation of the peptide linkers between class A repeats of the LDLR family [37]. Further research is required to understand the localization and functional specificity of these substrate proteins in different tissue cells.

## 4. Role of UDP-GalNAc: polypeptide N-acetylgalactosaminyltransferases in cardiovascular disease

In eukaryotic cells, around 80% of secreted and membrane-bound proteins undergo glycosylation by O-linked GalNAc. This process plays pivotal roles in cell development, signaling, interactions [38], adhesion[39], receptor-ligand binding [40], and interaction with pathogenic bacteria and viruses [41]. There is now substantial evidence confirming that patients with various CVDs, such as myocardial hypertrophy, heart failure, lipid disorders, coronary artery disease (CAD), and vascular calcification (VC), exhibit abnormal O-GalNAc changes.

## 4.1 GalNAc-Ts and the heart

### 4.1.1 GalNAc-T1 and GalNAc-T2 mediate O-glycosylation of proBNP

**Figure 2.**
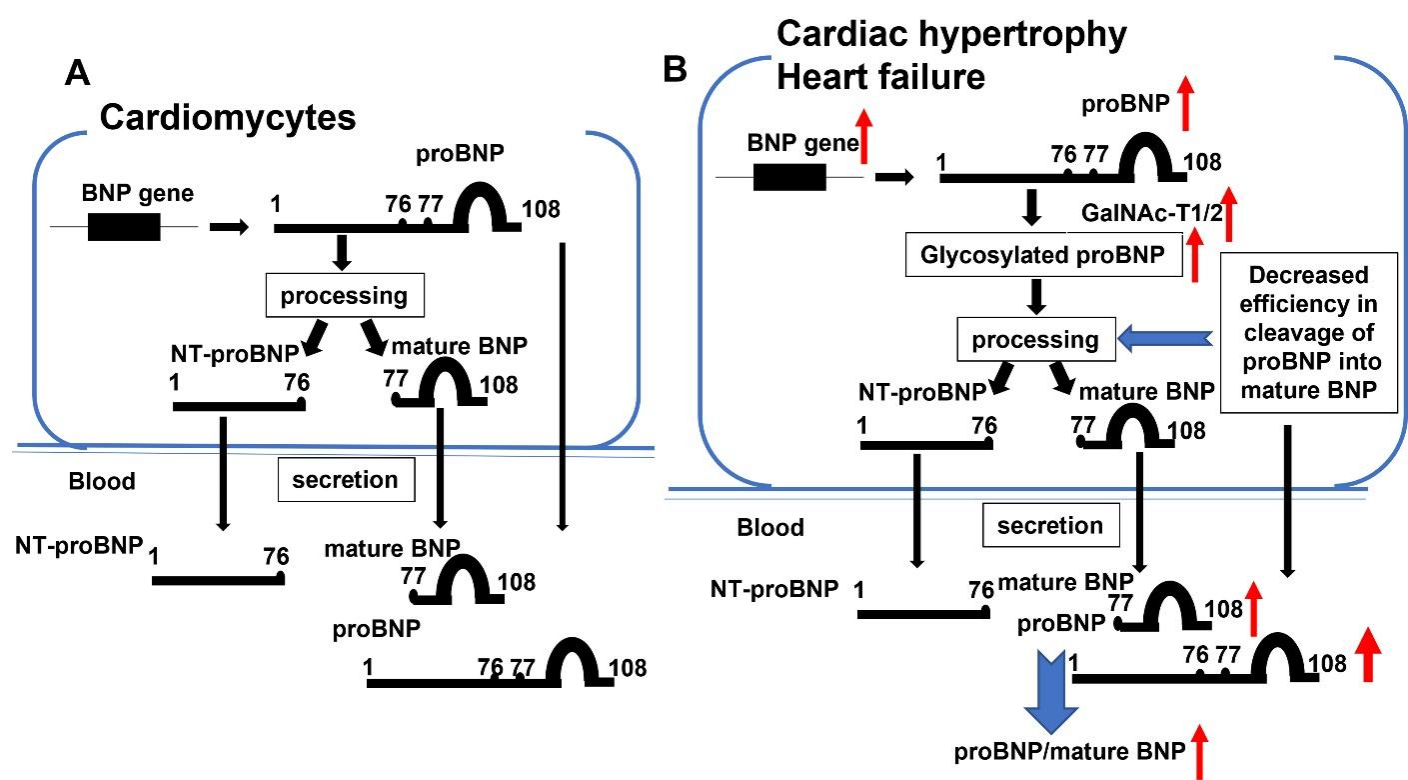
**Roles of GalNAc-T1 and GalNAc-T2 in cardiac hypertrophy and heart failure**. (**A**) In normal cardiomyocytes, glycosylation of proBNP at Thr71 prevents cleavage to form bioactive BNP-32 and NT-proBNP. (**B**) In patients with cardiac hypertrophy and heart failure, the elevated GalNAc-T1 and GalNAc-T2 mediate glycosylation of proBNP, which act cooperatively to inhibit the processing of proBNP, thereby increasing proBNP secretion by cardiac myocytes.

Brain natriuretic peptide (BNP) is a hormone that helps decrease systemic vascular resistance and central venous pressure while increasing natriuresis [42]. BNP is produced as a 108 amino acid prohormone, proBNP, which is in turn cleaved intracellularly to form the biologically active mature BNP (BNP-32) and inactive N-terminal proBNP (NT-proBNP) before their secretion[43]. In normal conditions, glycosylation of proBNP at Thr71 prevents cleavage to form bioactive BNP-32 and NT-proBNP (as shown in Fig. 2A). However, a study by Nakagawa et al[44] found that levels of GalNAc-T1 and GalNAc-T2 increased in HF patients. Then, GalNAc-T1 and GalNAc-T2 mediate glycosylation at two O-glycosylation sites in human proBNP, Thr48 and Thr71, which act cooperatively to inhibit the processing of proBNP, thereby increasing proBNP secretion by cardiac myocytes (as shown in Fig. 2B). Furthermore, the cardiac GalNAc-T1 and GalNAc-T2 expression was suppressed by microRNA (miR)-30, which is abundantly expressed in the myocardium of healthy hearts, but is suppressed in failing hearts. This miR-30-GALNT1/2 axis whose dysregulation increases the proportion of inactive proBNP secreted by the heart and impairs the compensatory actions of BNP during the progression of HF.

Another study [45] investigated the relationship between proBNP glycosylation, plasma NT-proBNP, and body mass index (BMI) in HF patients. They found the processing of proBNP is dysregulated in patients with HF, and increased BMI is associated with decreased concentrations of proBNP not glycosylated at Thr71 (NG-Thr71), the authors concluded that impairment of proBNP processing owing to glycosylation of Thr71 is relevant to reduce plasma concentrations of BNP and NT-proBNP observed in obesity.

### 4.1.2 GalNAc-T2 co-regulates the processing of β1AR and modulates its downstream signaling

Beta-adrenergic receptors (βARs) are a type of G protein-coupled receptors that activate intracellular signaling pathways by binding with agonists such as adrenaline and noradrenaline [46]. There are three subtypes of βARs (β1-, β2-, and β3-ARs), which are differentially expressed in the body. β1AR is the most abundant subtype in the heart[47] and is intensively studied because it controls physiological mechanisms that impact the pathogenesis of CVDs[48]. Goth et al [49] first demonstrated the existence of a regulatory interplay between site-specific O-glycosylation and proteolytic processing in the extracellular N terminus of β1AR, they found that GalNAc-T2 specifically O-glycosylates β1AR at five residues (Thr-28, Ser-37, Ser-41, Ser-47, and Ser-49) in the β1AR N terminus. In terms of mechanism, further research [50, 51] has shown that β1ARs are expressed in cardiomyocytes and other cell types as both full-length and N-terminally truncated species. The truncated β1AR species is formed as a result of an O-glycan-regulated N-terminal cleavage by ADAM17 at Arg31↓Leu32. However, O-glycan modification at Ser41 prevents ADAM17-dependent cleavage of the β1-AR N-terminus at Ser41↓Leu42. This prevents β1AR N-terminal cleavage and influences β1AR signaling to downstream effectors in cardiomyocytes.

### 4.1.3 GalNAc-T4 protects against cardiac hypertrophy through the ASK1 signaling pathway

Mitogen-activated protein kinase (MAPK) signal cascades play crucial roles in cardiovascular pathophysiology. The ASK family, including ASK1, ASK2, and ASK3, are key molecules in MAPK signal cascades and are activated by various stresses. A recent study by Zhang et al [52] showed that GalNAc-T4 was upregulated during the pathological process of cardiac hypertrophy. GalNAc-T4-KO mice demonstrated accelerated cardiac hypertrophy, dysfunction, and fibrosis. The in-depth mechanistic exploration clarified that GalNAc-T4 can directly bind to ASK1 to inhibit its N-terminal dimerization and subsequent phosphorylation, leading to a robust inactivation of downstream c-Jun N-terminal kinase (JNK)/p38 and NF-κB signaling. These results suggest that O-GalNAc glycosylation modifies normal valve development and cardiac function, and plays an important role in the development and progression of myocardial hypertrophy and HF. However, the detailed molecular relationship between GalNAc-T4 and the activation of ASK1 needs further clarification.

## 4.2 GalNAc-Ts and lipid disorders

### 4.2.1 Genetic mutation of the *GALNT2* gene associated with HDL-C levels

High levels of cholesterol in the blood can increase the risk of CVDs [53]. Normally, high-density lipoprotein (HDL) helps to protect against the oxidation of low-density lipoprotein (LDL). However, in certain conditions such as diabetes, inflammation, and oxidative stress, HDL changes in a way that makes it unable to protect against artery and inflammation problems [54]. A wide range of studies both in humans and animal models have pointed out that GalNAc-T2 plays a crucial role in shaping serum HDL-C and TG levels. A genome-wide association study (GWAS) has identified a common SNP, rs4846914, located in the first intron of the *GALNT2* gene, which is linked to lower GALNT2 expression in the human liver, is also associated with increased TG and decreased HDL-C levels[55, 56]. These observations validated GalNAc-T2 as a biological mediator of HDL-C levels. Furthermore, a clinical study suggests that a specific genetic mutation (rs12040273) is associated with higher levels of HDL cholesterol in the Chinese Han population [9].

### 4.2.2 GalNAc-T2 regulates ANGPTL3 cleavage *in vitro* cultured cells and *in vivo* mice

The angiopoietin-like protein 3(ANGPTL3) protein plays a crucial role in inhibiting the activity of endothelial and lipoprotein lipases, making it a promising target for drug therapies. ANGPTL3 specifically expresses in the liver and undergoes proprotein convertase processing (RAPR^224^↓TT) for activation, and the processing site contains two potential GalNAc O-glycosylation sites immediately C-terminal (TT^226^) [57]. A research study conducted by Schjoldager et al [57] discovered that the activation of ANGPTL3 is influenced by O-glycosylation, a process that is likely controlled by GalNAc-T2. They established that when GalNAc-T2 glycosylates Thr226 in a peptide with the RAPR224↓TT processing site, it blocks furin cleavage *in vitro*. Further study systematically characterized the cleavage of ANGPTL3 in cultured cells and *in vivo* mice [58]. The researchers found that endogenous ANGPTL3 is cleaved in primary hepatocytes and *in vivo* mice, and this cleavage can be blocked by GalNAc-T2 overexpression while suppressing GalNAc-T2 expression increases the cleavage of ANGPTL3 in mice dramatically. More recently, another study discovered that *Galnt2*^-/-^ mice have increased plasma ANGPTL3 protein levels. However, despite this increase, the *Galnt2*^-/-^ mice do not show significant changes in plasma triglycerides, either in the fasting state or after an oral fat load. This may be due to quantitative changes in other GalNac-T2 substrates [59].

### 4.2.3 GalNAc-T2 initiates mucin-type O-glycosylation of apolipoprotein-CIII

Apolipoprotein-CIII (apoC-III) is a type of glycoprotein found in blood. It is present on the surface of lipoprotein particles and is an important regulator of triglyceride metabolism [60]. Apo-CIII exists in four major proteoforms, a native peptide (CIII(0a)), and three different glycoforms containing a mucin-type core-1 O-glycosylation with zero (CIII(0b)), 1 (CIII(1), most abundant), or 2 (CIII(2)) sialic acids[61]. Recent studies have shown that GalNAc-T2 is responsible for initiating mucin-type O-glycosylation of apo-CIII in the Golgi apparatus [62]. Mutations in the *GALNT2* gene can cause rare congenital glycosylation disorders that affect apo-CIII glycosylation [36]. In addition, a study conducted by GWAS on the four apo-CIII proteoforms in people with and without type 2 diabetes [20] confirmed that the *GALNT2* gene plays a major role in the O-glycosylation of apoC-III. This alteration affects the function of apo-C III, which in turn changes the lipid profile, increasing the risk of type 2 diabetes.

### 4.2.4 GalNAc-T11 mediated O-glycosylation of LDLR-related proteins

The LDLR and related receptors are membrane-bound cell surface receptors important for the transport of diverse biomolecules across cell membranes and barriers [63]. Their functions are especially relevant for maintaining cholesterol homeostasis, and deleterious mutations in LDLR lead to decreased LDL catabolism and elevated levels of plasma LDL-cholesterol [37].

Previous studies have suggested that O-glycosylation of LDLR in the N-terminal domain is crucial for LDL binding and uptake [64]. In a study conducted by Pedersen et al [11], they identified the O-glycosylation sites of recombinant LDLR shed from SimpleCells and CHO wild-type cells. They find that members of the LDLR-related protein family share LDLR class A (LA) repeats (complement-like cysteine-rich ligand binding repeats) that assist in binding lipoproteins and other biomolecules. Moreover, the short linker regions between LDLR class A repeats contain an O-glycosylation site at position-1 of the first cysteine residue of most repeats, which is controlled by GalNAc-T11. In another study, Wang et al [10] found that GalNAc-T11 is responsible for the O-glycosylation at the Thr in the XXC6XXXTC1XX motif of linkers in LDLR and other receptors.

## 4.3 GalNAc-Ts and atherosclerosis

### 4.3.1 GalNAc-T1 and GalNAc-T11 involved in the O-glycosylation of chemokine receptors

Recruitment of leukocytes from the bloodstream into the vessel intima is a crucial step in initiating atherosclerosis and mainly occurs in the inflamed endothelium [65]. It is facilitated by a group of adhesive molecules and chemokine receptors, which are glycosylated proteins. A study by Verhallen et al [66] indicates that GalNAc-T1 and GalNAc-T11 are involved in the process of O-glycosylation of chemokine receptors. This process is important for fine-tuning and recognition of chemokine interactions with CCR5, which subsequently affects the signaling and functioning of the immune system.

### 4.3.2 GalNAc-T4 modifies the glycosylation of PSGL-1 and helps in the adhesion and transportation of monocytes

Adhesion molecules play a crucial role in the development of atherosclerosis. One such molecule is P-selectin glycoprotein ligand-1 (PSGL-1), which is responsible for selection and chemokine binding [67]. PSGL-1 is a transmembrane protein that appears as a disulfide-linked homodimer on the cell surface. The ectodomain of PSGL-1 is made up of a mucin-like region that is heavily O-glycosylated. This region extends the N terminus, which is functionally important for selectin binding [68]. A recent study [69] found that GalNAc-T4 participates in the synthesis of PSGL-1. Patients with acute coronary syndrome display an increase in GalNAc-T4 expression in their peripheral blood monocytes. Increased O-GalNAc glycosylation levels, which modify the glycosylation of PSGL-1, help in the adhesion and transportation of monocytes. Conversely, GalNAc-T4 knockdown inhibits P-selectin-induced activation of β2 integrin on the surface of monocytes, decreases monocyte adhesion under flow conditions with P-selectin stimulation, and suppresses monocyte transmigration triggered by monocyte chemotactic protein-1(MCP-1).

**Figure 3.**
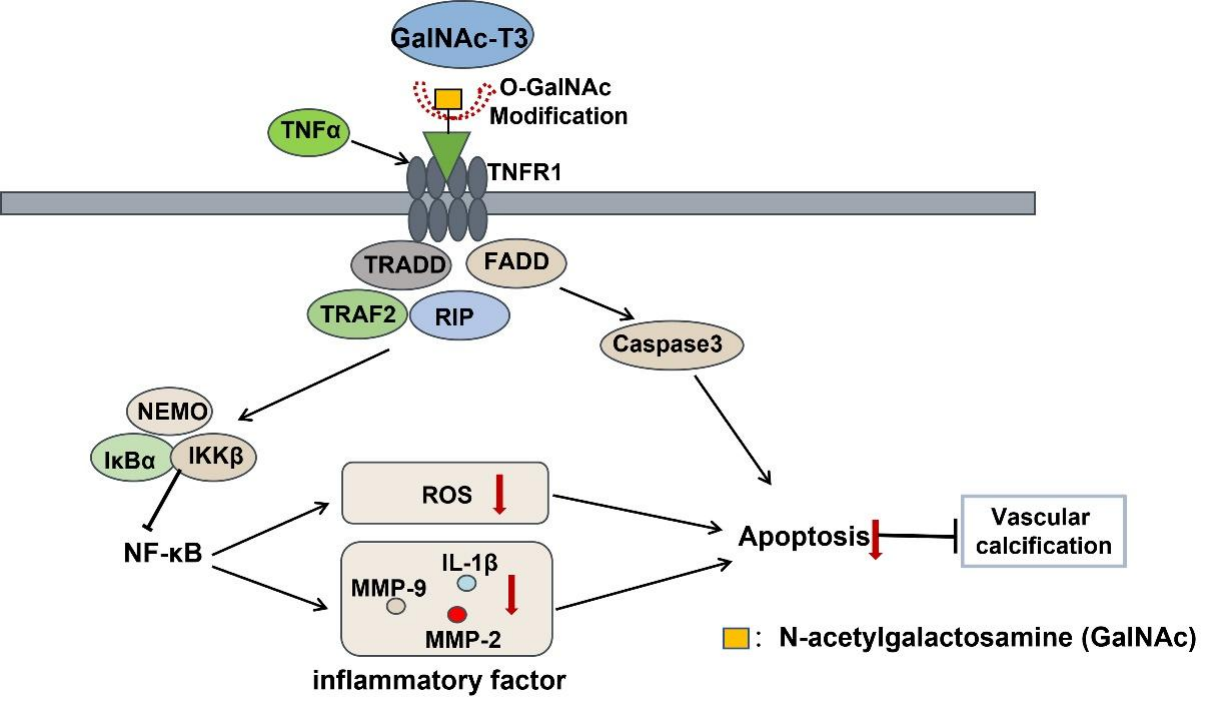
**Roles of GalNAc-T3 in vascular calcification**. GalNAc-T3 modified the O-GalNAc glycosylation of TNFR1 and inhibited its downstream NF-κB signaling pathway. As a result, GalNAc-T3 regulated oxidative stress and apoptosis of vascular smooth muscle cells, leading to amelioration of vascular calcification.

### 4.3.3 GalNAc-T3 protects against endothelial injury through the mitogen-activated protein kinase p38 pathway

Atherosclerosis is a major pathogenic mechanism of CAD and endothelial injury is recognized as an early event in the pathological process of atherosclerosis [70]. Our previous genome-wide gene expression analysis found the GalNAc-T3 expression was reduced considerably in CAD patients compared with control subjects. In the subsequent research, we investigated the relationship between single nucleotide polymorphism (SNP) in the *GALNT3* gene and CAD. The study found that two SNPs, rs13427924 and rs4621175, located in the intron region of the *GALNT3* gene, were associated with CAD [18]. It is worth mentioning that our previous research revealed GalNAc-T3 as a potential therapeutic target for CAD [15]. We have observed that knockout of the GalNAc-T3 in endothelial cells leads to increased cell apoptosis and activates the expression of matrix metalloproteinase 2 (MMP2) and MMP14 through the mitogen-activated protein kinase p38 pathway. This, in turn, results in damage to the endothelium [15]. However, the mechanisms involved in this process are yet to be uncovered.

### 4.4 GalNAc-Ts and vascular calcification

Vascular smooth muscle cell (VSMC) calcification is a risk factor for CVDs and a common complication of atherosclerosis and chronic kidney disease [71]. It is characterized by the pathological deposition of calcium and phosphate in blood vessels [72]. *In vitro* experiments confirmed that FGF23 is O-glycosylated through GalNAc-T3, which prevents its proteolysis and results in the secretion of biologically active intact FGF23[73]. Inactivating GalNAc-T3 mutations makes FGF23 susceptible to proteolysis, thereby reducing circulating intact hormone levels. This leads to lower hormone levels and causes hyperphosphatemic familial tumoral calcinosis [74]. Our previous study has shown that GalNAc-T3 helps prevent phosphate-induced calcification in VSMCs. This is achieved by enhancing active FGF23 and inhibiting the Wnt/β-catenin signaling pathway [16]. Additionally, our team has reported for the first time that GalNAc-T3 inhibits VC by reducing oxidative stress, inflammation, and apoptosis of VSMCs [17]. Regarding the underlying molecular mechanisms, our data suggests that GalNAc-T3 protects against VC by O-GalNAc glycosylation of tumor necrosis factor receptor 1 (TNFR1), which suppresses its downstream NF-κB pathway, thus protecting against VC (as shown in Fig. 3).

**Table 1:** Glycosylation substrates of GalNAc-Ts in cardiovascular disease.

GalNAc-Ts	Substrates	Pathophysiological conditions	Mechanisms
**GalNAc-T1/GalNAc-T2**	proBNP	cardiomyocytes	increasing the ratio of proBNP/BNP secretion
**GalNAc-T2**	β1AR	cardiomyocytes	prevented β1AR N- terminal cleavage and influenced β1AR signaling to downstream effectors
**GalNAc-T2**	ANGPTL3	lipid disorders	blocked proprotein convertase processing for ANGPTL3 activation
**GalNAc-T2**	Apo-CIII	lipid disorders	affects apo-C III function
**GalNAc-T11**	LDLR	lipid disorders	increases LDLR lipid binding and uptake
**GalNAc-T1/GalNAc-T11**	CCR5	leukocytes recruitment	O-glycosylation of chemokine receptors
**GalNAc-T4**	PSGL-1	monocytes adhesion and transportation	modifies the glycosylation of PSGL-1
**GalNAc-T3**	FGF23	phosphate-induced calcification in vascular smooth muscle cells	enhancing active FGF23

## 5. Conclusions and perspectives

Mucin-type O-glycosylation is a process that occurs in different organs of the human body and is associated with a variety of pathophysiological conditions in CVDs. In this review, we aim to summarize the link between mucin-type O-glycosylation and CVDs, highlighting the significance of the GalNAc-Ts in disease development and progression through glycosylation of various substrates. The identified glycosylation substrates of GalNAc-Ts in cardiovascular disease are listed in Table 1. For instance, GalNAc-T1 and GalNAc-T2 are responsible for the glycosylating of human proBNP, which contributes to the increased secretion of proBNP by attenuating its processing. GalNAc-T2 plays a crucial role in the regulation of HDL cholesterol levels, affecting lipid metabolism. On the other hand, GalNAc-T11 regulates the glycosylation of the LDL and VLDL receptors, which substantially increases their lipid binding and uptake. Nonetheless, these findings still require validation through clinical evidence. Newly developed techniques allow us to better understand the mechanism of O-GalNAc glycosylation and pave the way for further research on glycosylation. Genetically engineered cells termed SimpleCell have been used to identify O-glycosylation sites [75]. Mass spectrometry instrumentation and intelligent computing have proven helpful in identifying O-glycosylation sites in a sample [76]. A recent study described an initial *in vivo* map of the O-glycoproteome and a practical approach to quantitatively map O-glycosylation sites in complex samples such as tissue extracts and biological fluids [77]. Using liver samples isolated from wild-type and *Galnt2*-null mice, they identified hubs of *Galnt2*-modified glycoproteins which may account for lipid and metabolic dysregulation observed in patients with congenital disorders of O-glycosylation.

More work is still needed to fully understand the roles of GalNAc-Ts in CVDs. It's important to consider some crucial questions related to the relationship between GalNAc-Ts and CVDs. Firstly, which protein substrates change during the development of CVDs-are they increased or decreased? Secondly, what are the effects of these changes, and are they harmful or beneficial? Thirdly, which specific loci of protein O-GalNAc glycosylation are involved, and how do site-specific O-glycans regulate the target protein's function? Answering these questions is crucial to determining the potential of GalNAc-Ts as a treatment for CVDs. In addition, small molecule inhibitors of GalNAc-Ts are valuable tools for studying glycan functions. Studies have identified selective inhibitors of GalNAc-T3 [78] and two compounds that broadly inhibit Golgi-localized glycosylation processes [79]. With further studies into the mechanism of action for these compounds, they can potentially serve as therapeutic targets of CVDs.

To sum up, recent studies have shown a link between GalNAc-Ts and CVDs. Further investigation into these molecules and their roles could provide new ideas for the prevention and treatment of CVDs.
